# The effect of occupational therapy services on hospital readmission for patients with cancer in acute care settings: a retrospective data analysis

**DOI:** 10.1007/s11764-024-01620-4

**Published:** 2024-05-31

**Authors:** Christine C. McNichols, Alicia K. Peterson, Stacey Reynolds

**Affiliations:** 1 Occupational Therapy, Virginia Commonwealth University, 900 E. Leigh St, Richmond, VA 23298 USA; 2 Division of Research, Kaiser Permanente Northern California, Pleasanton, CA USA

**Keywords:** Occupational therapy, Readmission, Hospital, Acute care, Cancer

## Abstract

**Purpose:**

This study examined how the use of occupational therapy services affected the likelihood of hospital readmission within 30 days for patients with cancer diagnoses.

**Methodology:**

This was a retrospective observational study. Patient medical records were analyzed from a National Cancer Institute Hospital over a 5-year period with a sample size of 6614 patients included for analysis in an unadjusted logistic regression model and 1920 patients analyzed in an adjusted logistic regression model. Various factors, including the use of occupational therapy services as well as individual factors such as pain levels, cancer stage, and living environment, were considered in relation to readmission status. Logistic regression analyses were used to assess the provision of occupational therapy service’s association with 30-day hospital readmissions.

**Results:**

Patients who received occupational therapy services had a statistically significant decrease in their risk of a 30-day hospital readmission compared to patients with cancer who did not receive occupational therapy services. In an unadjusted analysis, patients with cancer who had occupational therapy services were 33.5% (OR = 0.665) less likely to be readmitted within 30 days compared to a patient who did not have occupational therapy services (*p* **<** 0.001). In an analysis after adjusting for patient health–related factors, patients with cancer who had occupational therapy services were 22.2% (OR = 0.778) less likely to readmit to a hospital compared to a patient who did not have occupational therapy services (*p* **<** 0.046).

**Conclusion:**

The results of the study are intended to contribute to the body of knowledge on the benefits of occupational therapy services on an individual as well as a health systems–based level for patients with cancer diagnoses while hospitalized.

**Implications for Cancer Survivors:**

The knowledge of the utility of occupational therapy services for patients with cancer diagnoses while in the hospital can assist providers, patients, and hospital leadership in understanding some of the potential benefits for patient care and healthcare systems at large while seeking to avoid the deleterious effects from a hospital readmission.

**Supplementary Information:**

The online version contains supplementary material available at 10.1007/s11764-024-01620-4.

## Introduction

Twelve to seventeen billion dollars is spent annually in the United States on healthcare expenditures resulting from unexpected hospital readmissions [1]. A hospital readmission is when a patient returns to a hospital within a certain time period (generally 30 days) after the patient is discharged [2]. Within the last decade, hospital readmission rates have become an increasingly important metric for facilities as funding and reimbursement may be related to readmission rates both in direct and indirect ways [3]. Occupational therapy is a rehabilitation discipline that works to improve independence with functional abilities in those recovering from illness as well as provides patients and family members recommendations for safe discharge. Scant information is available as to the effects that occupational therapy services may have on patient readmission status, and there is an absence of data specific to patients with cancer diagnoses. This research study investigated the relationship between occupational therapy services and readmission status in patients with a cancer diagnosis. It was hypothesized that occupational therapy services would reduce the likelihood of patient readmission when compared to those patients with cancer who did not receive services.

## Background

### Hospital readmissions and why they matter

New legislative changes in the twenty-first century, including the Patient Safety and Quality Improvement Act of 2005 and the Patient Protection and Affordable Care Act (2009), contributed to changes in the Center for Medicare and Medicaid Services (CMS) reporting requirements as well as increased transparency required by hospitals in the reporting of discharges [3]. Subsequently in 2012, CMS instituted the Hospital Readmissions Reduction Program (HRRP) as a Medicare value-based program that adjusts a facility’s funding based upon unplanned re-admissions for six diagnoses including acute myocardial infarction (AMI), chronic obstructive pulmonary disease (COPD), heart failure (HF), pneumonia, coronary artery bypass graft surgery (CABG), and elective primary total hip arthroplasty and/or total knee arthroplasty (THA/TKA) [4]. Medicare is governmental insurance in the United States for people 65 years or older and younger individuals with disabilities [5]. Under this new system, hospitals with excessive readmissions see a reduction in their payments for unplanned readmissions within 30 days after discharge [4]. Fines for high readmission rates can be large with readmission penalties from 2016 causing an expected loss nationwide of over half of a billion dollars in 2017 alone [6]. While cancer is not included as one of the six core HRRP diagnoses, patients with cancer may be admitted to the hospital with a diagnosis such as AMI, COPD, or HF. Therefore, readmission metrics are salient for the cancer population.

While there are far-reaching financial impacts on healthcare and hospital systems, patient-centric effects from additional readmissions are also of grave concern. With unexpected hospital admissions, patients are at increased risk of hospital-acquired infections and deconditioning, as well as increased risk for medical errors [1]. Patients with a cancer diagnosis may be immunocompromised for a variety of reasons including their age, a metabolic disorder, their cancer typology, or cancer treatments [7] which place them at a greater risk for infections. Additionally, in-hospital mortality has been found to be higher for patients who have been readmitted when compared to those who have not [3]. As such, it is vital to ensure that the readmission of patients with cancer occurs as seldom as possible.

### Common readmissions causes

Approximately 14% of all patients are readmitted to a hospital within 30 days of their discharge [8], and almost 20% of all patients with Medicare have had a hospital readmission within 30 days [9]. In contrast, approximately 16% of patients with cancers of solid tumors [10] and 27% of patients with late-stage cancer have been found to be readmitted to a hospital within 30 days of discharge [11]. Additionally, 16%–25% of patients will be readmitted to a hospital within 30 days after major cancer surgery [12]. This data highlights the increased risk cancer patients have for hospital readmission and the need for services to help reduce this risk.

Interestingly, it is estimated that one-quarter of 30-day hospital readmissions are potentially avoidable and preventable [13]; with estimations that if 10% of avoidable readmissions were prevented, Medicare savings would be $1 billion dollars [9]. However, further research is needed to determine what specific factors might prevent hospital readmissions. Some of the reasons that patients may be readmitted to a hospital quickly may include medication errors, failures to plan for equipment needed in the home, deficits in care, and improper preparation of the patient and/or the family for the patient’s care upon leaving the hospital [2], all of which may impact a safe discharge for patients with cancer.

### Occupational therapy service provision

Patients with cancer most frequently exhibit functional limitations in areas of personal hygiene (self-care), walking, and transfers [14]. Prior research in the general patient population, while not cancer-specific, has seen increased readmission rates for patients who have unmet activity of daily living (ADL) needs [15], and a patient’s functional status has been found to affect the likelihood of readmission to a hospital [16]. Greysen and colleagues [17] specifically found a progressive increase in the risk of readmission as one’s functional impairment increases, emphasizing that functional deficits may impact safety and independence upon hospital discharge.

Occupational therapy is a rehabilitative discipline that engages clients in functional therapeutic tasks in order to enhance or enable participation. Occupational therapy services are ordered by medical providers, which include physicians and nurse practitioners [18], in the hospital setting. Many patients in the United States receive occupational therapy services during a hospital stay with approximately 30% of all occupational therapy jobs found in hospital settings [19]. Cost and charges to a patient may vary based on the patient’s hospital status and payor source [20].

Occupational therapy practitioners are skilled practitioners who are able to analyze and address self-care and mobility deficits, as well as minimize the functional effects of disease, injury, and/or disability [16]. Services can be focused on the promotion of health and wellness in addition to rehabilitative needs as they may impact daily life. Occupational therapy practitioners are also well poised to provide compensatory strategies in addition to equipment and environmental recommendations to enhance a patient’s functional independence [21]. Patients who may benefit from occupational therapy services may have needs relating to self-care, home management, functional mobilization, cognition, range of motion, and other physical or psychosocial areas which can impede an individual’s livelihood. Functional and social needs can be important factors in a patient’s readmission likelihood and are able to be addressed by occupational therapists [22]. By focusing on addressing patients’ functional needs during the acute care stay and providing safe discharge recommendations, occupational therapy practitioners have the potential to reduce readmission rates in patients with cancer.

Research to date suggests that occupational therapy services can improve patient function and reduce readmission rates in various diagnostic groups [23]; however, no research to date has examined this phenomenon with cancer patients specifically. Freburger and colleagues [24] found that following a stroke, patients who received outpatient therapy (occupational and/or physical) in the first 30 days after their discharge were less likely to have a hospital readmission within 30 days when compared to those who had not received therapy services. For patients hospitalized with pneumonia, it was found that when patients had increased levels of self-care independence, there was a reduction in the odds of readmission [25]. Moreover, Edelstein and colleagues [26] found that when patients were in the hospital with one of the six HRRP-qualified diagnoses, those who received occupational therapy services during their hospital stay with a higher frequency of services had lower odds for a hospital readmission. The same study found that of those patients who were readmitted to the hospital, those individuals had a greater likelihood of not having received activities of daily living (ADL) or self-care education during their hospital admission [26].

In summary, research to date suggests that some hospital readmissions are preventable and that occupational therapy services may play a key role in reducing these readmissions. Patients with cancer are specifically at high risk of readmissions, and research is limited in this population. The current study will help fill these gaps by determining how occupational therapy is associated with readmission status for this patient population.

### Study objectives

The purpose of this study was to examine the potential association between occupational therapy services for patients with a cancer diagnosis and their likelihood of having a hospital readmission within 30 days from hospital discharge. The aim of the research was to examine potential relationships between patient factors, which included cancer stage and type, in addition to receipt (or not) of occupational therapy services and hospital readmission. The research questions were as follows:

1. Do patients with cancer who receive occupational therapy services in an acute care setting have a difference in the likelihood of readmission within 30 days status post-discharge than patients who did not receive occupational therapy services?
2. Do patients with cancer who receive occupational therapy services in an acute care setting have a difference in the likelihood of readmission within 30 days status post-discharge than patients who did not receive occupational therapy services after adjusting for sex, race, ethnicity, age, admitting diagnosis, cancer type, cancer stage, discharge location, living situation, pain levels, and insurance type?

The primary hypothesis for the study was that patients with cancer who received occupational therapy services in an acute care setting would have a lower likelihood of readmission within 30 days status post-discharge compared to patients who did not receive occupational therapy services, even after accounting for demographic and diagnostic factors, based upon prior research for other patient populations [23–26].

## Methods

### Study design and data selection

Pre-existing data was extracted from electronic health records at Virginia Commonwealth University’s Massey Cancer Center in Richmond, Virginia (referred to as Massey henceforth) for retrospective analysis. As a large urban medical center and a National Cancer Instituterecognized facility, Massey provides inpatient and outpatient services to patients with cancer from a diverse and expansive geographic area [27]. The sole focus of this study was on inpatient (i.e., acute care) occupational therapy services with an investigation into the potential association between occupational therapy services and hospital readmissions. Data was extracted for patients who received services at Massey from the dates of January 1, 2015, through January 1, 2020. The selected sample dates were chosen to prevent any potential impact from healthcare changes (e.g., COVID-19) or major healthcare regulatory actions while also ensuring a substantial enough sample size to support a proper power study. In order to create a sample size necessary for the statistical analyses, G*Power [28] was used with a two-tailed logistic regression model, which will be able to see if a statistically significant difference exists between odds using the Wald statistic (displayed in Tables 1 and 2), with an odds ratio of 1.3 with an err prob (α) set at 0.05 and a desired power (1-β err prob) set at 0.95, with a sample size of 1188 participants necessary for study inclusion.

**Table 1:** Crude logistic regression results for readmission and occupational therapy (OT) services

Variable	OR	*p*	95% CI: lower	95% CI: upper	*B*
Received OT services in hospital*	0.665	< 0.001	0.586	0.753	−0.408

Note. *p* < 0.05 is considered a significant difference

*OR:* odds ratio

*OT services reference to OT services not received

**Table 2:** Logistic regression results of OT services and odds of hospital readmission after covariate adjustment

Variable	OR	*p*	95% CI: lower	95% CI: upper	*B*
Received OT services in hospital*	0.778	< 0.046	0.608	0.996	−0.251

Note. Model adjusted for sex, race, ethnicity, age at visit, admission diagnosis as cancer, cancer type, cancer stage, living situation prior to admission, earliest pain score, last pain score, insurance type, and discharge placement. *p* < 0.05 is considered a significant difference

*OR: o*dds ratio

*OT services reference to OT services not received

### Ethics

After receiving institutional review board (IRB) approval from VCU, the data collection location and the data extraction occurred without the inclusion of any patient identifiers. Massey’s health informatics and information technology teams supported data extraction and preservation from Cerner, which was VCU’s electronic health record during the intended timeframe.

### Participants

Data extraction was based on pre-selected inclusion and exclusion criteria. To focus the scope of this research, based on national frequency as well as center-specific frequency, six of the most prevalent cancer types were included: breast, blood/hematologic, colorectal/gastrointestinal, gynecologic, lung/respiratory, and prostate/genitourinary [29]. Inclusionary criteria included individuals who were at least 18 years of age with a cancer diagnosis and who had a qualifying hospital stay (a minimum of a 3-day or longer hospital admission to allow for adequate time for the completion of an occupational therapy evaluation and subsequent treatment sessions if needed). While the patient population of interest in the current study was patients with a cancer diagnosis, the cancer diagnosis did not have to be the primary admitting diagnosis for their hospitalization. For example, a patient who has cancer that has metastasized to their brain could have balance disturbances that would contribute to a fall requiring hospitalization. While the fall may be a result of the cancer, the likely admitting diagnosis would be “fall.” To not preclude patients with a myriad of admitting diagnoses, having cancer was a requirement for inclusion within the research study as well as having received cancer services through Massey Cancer Center; however, cancer as the primary admitting diagnosis was not necessitated for inclusion. Subjects were only included if they had a consistent living environment before their hospital admission with acceptable options including living at home alone, in the home with others, in long-term supports and services (LTSS), or others (a self-select option chosen by a patient). The term LTSS in the “lives with” grouping was used to include patients who resided at home with caregiver support (non-habitable), in an assisted living facility, or in a nursing home. This allowed for a potential focus on discharge planning and recommendations to assist in returning to a prior living environment. If a patient was unhoused or if a patient was admitted for a pre-planned elective surgery (e.g., a total knee replacement), they were excluded from this study.

### Outcome variable

The main outcome of interest was a hospital readmission within 30 days (yes/no). Statistical analyses were performed to determine the contributions of occupational therapy services to the primary variable of hospital readmission; additional analyses explored this relationship after adjusting for demographic and diagnostic covariates.

### Predictor variables

Variables of interest in this study were analyzed to determine any potential effects on a patient’s odds of a hospital readmission. Our primary predictor of interest was occupational therapy service utilization, a categorical (yes/no) variable indicated by the presence (or absence) of at least one occupational therapy code billed in the patient’s account.

Various factors had the ability to influence both hospital readmissions and occupational therapy services and were included in the study as possible covariates and were dummy-coded for analyses, and the largest represented category within the variable was used as the reference category. Risk factors in combination with process factors were jointly referred to as covariates within the statistical model and were combined in order to attempt to ascertain any potential relationship to the outcome variable. Cancer type and stage as well as a patient’s discharge location were extracted from Massey’s database in order to determine any potential effect on the likelihood of readmission. Insurance type was included to ascertain if any insurance types contributed to the likelihood of readmission. The insurance-type categories were commercial insurance (refers to coverage by a private entity), corrections (insurance from the correctional system), indigent (patients with income below the Federal poverty line), Medicaid (state insurance program for patients who meet certain medical or financial criteria), Medicare (governmental insurance for people 65 or older and younger individuals with disabilities), military (those with military based insurance), other (any insurance that did not meet any of the other six categories), and self-pay (where the individual would pay for services themselves without insurance) [5, 30. Additionally, a patient’s race, ethnicity, age (years), gender, cancer as the admitting diagnosis (yes, no), and living environment were included for analysis. In addition, pain levels during sessions were included since uncontrolled pain has been found to have a negative effect upon quality of life and lead to potential readmissions [31]. A summary of key predictor variables is presented in Table 3. 

**Table 3:** Variables

Variable	Category
OT services	Nominal/categorical
Readmission within 30 days	Nominal/categorical
Cancer type	Nominal/categorical
Lives with	Nominal/categorical
Admitting diagnosis	Nominal/categorical
Cancer stage	Nominal/categorical
Gender	Nominal/categorical
Race	Nominal/categorical
Ethnicity	Nominal/categorical
Age	Scale/continuous
Pain at baseline	Scale/continuous
Pain at discharge	Scale/continuous
Insurance	Nominal/categorical
Discharge location	Nominal/categorical

*Note. OT:* occupational therapy, *LTSS:* long-term services and supports, *AMA:* against medical advice

### Data extraction and cleaning

Data extraction occurred with the assistance of the Massey information technology team and was assessed for missing data. The data was cleaned with variables removed for coding inconsistencies [32]. Complete case analysis was used whereby missing data in any patient category removed the patient from analysis in the study. Descriptive statistics were run on predictor and outcome variables to assess distribution, measures of central tendency, measures of variability, and skewness. No outliers were present within the data.

Before analysis, statistical assumptions were checked and met. In order to have independence of observations, only one admission per patient was able to be included for analysis. In place of a patient’s medical record number (MRN), a patient identification number (ID) was created to protect patient privacy by Massey. The patient’s ID number was then used to assess the number of admissions during the selected time frame. The first hospital admission was retained for statistical analysis and then the remaining admissions for a selected individual were removed from the data.

For research question one, there were 12,159 patient observations who initially met the inclusion and exclusion criteria for analysis. In order to have independence of events, the number of total patients was reduced to 6,614 to encompass only one unique visit (first admission) per unique patient based on the patient identification number.

For research question two, the data was cleaned and refined according to the complete case analysis. Initially, there were 12,159 unique admission cases (number of patients) but 19,113 total observations due to multiple observations that were attributed to each individual patient. As such, further explanation of the refinement process discusses observation numbers and not patient numbers. There were 9955 observations that were removed from the analysis as a result of not having a cancer stage and 107 observations that were removed due to missing race specification (unknown-unable to communicate or unknown-patient refusal). There were 59 observations removed due to missing ethnicity (unknown-patient refusal, unknown-unable to communicate, or N/A-outreach use only), and there were 8 observations removed due to a lack of an appropriate cancer type (designation: all others). Lastly, there were 3471 observations without any home information regarding the living situation, with 215 without the earliest pain score and 130 observations with no final pain score. After removing those with missing data, the sample was reduced to 5168 individual patient admissions (including multiple admissions across the timeframe for any particular patient). As such, 3248 cases were removed that were subsequent admissions, and the number of participants was further reduced to a final sample size of 1920 individual patient admissions with the selection of the patient’s first admission within the 5-year period to keep the independence of observations. The first admission was selected to reduce any potential mitigating effects that could arise from disease progression, such as could occur with a patient’s final admission, during the selected time period.

### Statistical analyses

To determine if receipt of occupational therapy services explained the likelihood of being readmitted within 30 days after discharge (research question one), the independent variable (IV) of receipt of occupational therapy services and the dichotomous dependent variable (DV) of hospital readmission were analyzed within the context of a crude logistic regression model using SPSS v28. The alpha level was set at 0.05. It was planned that if the significance level was less than 0.05, then the null hypothesis would be rejected.

For question two, an adjusted logistic regression analysis was used to assess if occupational therapy services affected the odds of readmission after adjusting for demographic and diagnostic factors. Categorical independent variables included occupational therapy services received (primary IV), cancer type, who the patient lived with, admitting diagnosis, cancer stage, gender, race, ethnicity, insurance, and discharge location; these categorical variables were dummy-coded. Additional independent variables were continuous numeric variables (i.e., age, pain at baseline, and pain at discharge) and were subsequently entered into the model. Alpha was set at 0.05 with the intention that if *p <* 0.05, then the null hypothesis would be able to be rejected.

## Results

Within the sample of 6614 patients included in the analysis for research question one, 18.7% of discharged patients were readmitted to the hospital within 30 days, and 81.3% of patients were not readmitted. Of all patients, 49.4% of patients received occupational therapy services during their inpatient hospital admission, and 50.6% of patients did not, which served as the reference group.

To address the question of whether patients with cancer who received occupational therapy services in an acute care setting had a difference in the likelihood of readmission within 30 days status post-discharge than patients who did not receive occupational therapy services, the results from the crude logistic regression model found that patients who had received occupational therapy services during their hospital stay had 0.665 (95% CI: 0.586, 0.753) the odds of readmission compared to those who did not receive services (*p* < 0.001). There was a statistically significant difference in the readmission risk when comparing the two groups. Results are shown in Table 1.

In reference to research question two, an adjusted logistic regression model was performed to ascertain if, after adjusting for various health-related factors, there was any effect of occupational therapy services upon readmission status. The frequency counts of the health-related factors used in the analyses can be found in Table 4. Some selections include approximately 40% of the patients within the sample did not enter the hospital as a direct result of their cancer diagnosis; stage IV cancer was the most prevalent cancer stage (45%), and Medicare comprised the largest group for payor source (44%). Of the 1920 individuals included in this analysis, 53.9% did not receive occupational therapy services, while 46.1% did receive occupational therapy services. Within this sample, 24.7% of patients had a readmission to the hospital within 30 days, while 75.3% of patients were not readmitted within 30 days of their discharge.

**Table 4:** Demographics

Gender	
Male	51.6%
Female	48.4%
Ethnicity	
Not Hispanic	97.7%
Hispanic	2.3%
Cancer as admitting diagnosis	
Cancer not admitting diagnosis	40.3%
Cancer as admitting diagnosis	59.7%
Cancer stage	
Stage I	15.6%
Stage II	18.2%
Stage III	21.4%
Stage IV	44.8%
Insurance type	
Commercial	29.4%
Corrections	0.9%
Indigent	4.6%
Medicaid	18.5%
Medicare	44.4%
Military	0.4%
Other	1.1%
Self-pay	0.7%
First pain score	
0	44.9%
1	0.9%
2	4.5%
3	4.3%
4	6.6%
5	6.2%
6	6.7%
7	6.2%
8	8.7%
9	3.8%
10	7.2%
Race	
White	53.4%
Black	41.5%
Asian	1.3%
Other	3.8%
Cancer type	
Breast	8.7%
Blood/heme	20.1%
Colorectal/gastrointestinal	35.6%
Gynecologic	5.9%
Prostate/genitourinary	8.6%
Respiratory	21.1%
Living situation	
Home alone	21.0%
Home with others	74.0%
LTSS	3.8%
Living-other	1.2%
Discharge location	
Home	52.7%
Rehabilitation facility	11.7%
Home with support	16.5%
Expired	9.2%
Hospice	8.8%
AMA	0.5%
Additional hospital care	0.6%
Last pain score	
0	58.0%
1	1.6%
2	7.1%
3	5.4%
4	7.1%
5	5.0%
6	6.1%
7	4.8%
8	3.1%
9	1.1%
10	0.7%

*Note. LTSS: *long-term services and supports, *AMA: *against medical advice

The adjusted logistic regression model demonstrated a significant association between occupational therapy services and the odds of a hospital readmission. The results from the adjusted logistic regression found that patients who had received occupational therapy services during their hospital stay had 0.778 (95% CI: 0.608, 0.996) the odds of readmission compared to those who did not receive services (*p* = 0.046) (Table 2). There was a statistically significant difference in the readmission risk when comparing the two groups. All parameters for the fully adjusted logistic regression model are included in Supplement Table S1.

## Discussion

### Aim, research questions, and hypotheses

The aim of the study was to examine the receipt of occupational therapy services and their association with the odds of hospital readmission. The null hypothesis was that the receipt of occupational therapy services for patients with a cancer diagnosis would not result in any change in the likelihood of hospital readmission within 30 days from discharge when compared to those patients who did not receive occupational therapy services. Based on our statistical findings, this hypothesis was rejected given that there were significantly significant differences in the odds of readmission for patients who received occupational therapy services during their hospital stay when directly compared to those patients who did not receive occupational therapy services. The null hypothesis was also rejected upon further statistical analysis with the use of the adjusted logistic regression. After adjusting for 13 different healthcare factors of physiological and societal factors, the receipt of occupational therapy services still significantly decreased the odds risk for readmission and had a protective association.

### Clinical relevance

The findings in the crude and adjusted logistic regression are clinically relevant for patients, practitioners, and healthcare systems. As readmissions have the capacity to affect patients physically and psychologically, as well as impact hospital systems monetarily, a reduction in the risk of readmitting to a hospital is important on many levels. On a patient-centric level, a decreased risk of a hospital readmission reduces the risk of negative patient outcomes such as hospital-acquired infections, deconditioning, medical errors, and death [1, 3]. On a healthcare systems level, a healthcare entity may see fiscal repercussions from a patient readmitting within 30 days from payments from Medicare. Interestingly, patients with Medicare comprised the largest payor group in our current study, suggesting the results are particularly relevant to the Medicare population and those who serve them.

Finding that occupational therapy services reduced the rate of readmission risk and remained statistically significant when adjusting for a myriad of factors supports prior findings that suggest that occupational therapy services are protective against hospital readmission risk [23–25]. As patients with cancer diagnoses can have extensive physical impairments, occupational therapy services provide an opportunity for patients to receive skilled individualized care to regain strength, train in safe functional mobility, and receive equipment and environmental recommendations for a safe return to a prior living environment. Occupational therapy is also unique in that it can address the cognitive and psychosocial changes that often accompany cancer diagnoses. By addressing concerns before a discharge from the hospital, occupational therapy services can prepare clients to return home even with new or advanced physical, psychosocial, or cognitive changes. Therefore, patients with cancer who receive these services may have less reason to return to the hospital quickly. The findings in this study substantiate the need for the provision of occupational therapy services and contribute to the formation of the knowledge base that occupational therapy services can assist in the prevention of hospital readmission. These findings may affect physicians and other healthcare providers in their analysis of patient needs during an admission ensuring patient safety and the provision of evidence-based clinical care.

### Limitations and future directions

This study sought to discern whether cancer patients who received occupational therapy services during their hospital stay had a lower likelihood of a hospital readmission when compared to patients with cancer who were not referred for occupational therapy services during the collected timeframe. The possibility exists that patients with cancer who are referred for occupational therapy services may inherently be different than patients who are not referred for occupational therapy services during a hospital admission. For example, patients *not* referred for occupational therapy services may not present as having any functional needs (no perceived benefit from services), or as not having the potential to benefit from rehabilitation services (e.g., they are dependent at baseline in care and mobility). Alternatively, patients who are referred for occupational therapy services during their hospital stay may lie between these two extremes and have functional deficits in combination with the perceived possibility for restoration of functional status. While no research currently is available on referral patterns for patients with cancer diagnoses in acute care settings, we do acknowledge that this is a possible study limitation. If the patient groups were different enough at baseline, then this would impact the study’s internal validity. We acknowledge this limitation and attempted to control this by comparing groups at baseline (evaluation) on factors such as cancer type, cancer stage, age, and baseline pain levels. Future studies should look to consider prospectively evaluating all patients with cancer for functional status at baseline (admittance to a hospital) and whether or not an occupational therapy referral was received; this would allow for group comparisons on factors such as self-care status and functional mobility. To better ensure reliability within the data, there was consistency in the analysis of only the first patient visit, accuracy checks with additional members of the research team, and complete case analysis. Future research could also look to analyze whether specific patient health–related factors had any effect upon the initiation of an occupational therapy order being placed or being performed in order to test for differences in service provision. Additionally, the patient sample was taken from one hospital system which may impact the generalizability of the results. Future studies may look to involve patient data from several hospital systems including variation for size, trauma level, profitability, and geographic location to see if differences exist between systems.

Ethnicity as well as patient-selected racial classifications were adjusted for within the statistical analyses of this study; however, future studies may seek to understand how health disparities may be present in occupational therapy service provision for patients with cancer diagnoses, potentially moderating the association. Understanding the referral process for occupational therapy services, or barriers that may exist to the ordering and execution of service, would be avenues to explore. Additionally, future studies may look at outcomes derived from occupational therapy services within various racial, ethnic subgroups, or social economic status groups as pertinent avenues for patients with cancer diagnoses.

Future studies could further analyze cancer-specific subgroups or cancer stages to see if any differences exist between patients at various cancer stages or typologies. This study pertained to six cancer types based upon frequency and prevalence nationally and within the institute of study; however, future studies could analyze additional cancer types both conjointly as well as in isolation to the effects of occupational therapy services. Additionally, specifics of occupational therapy service provision, occupational therapy frequency, and the subsequent billing codes which had the largest effect on readmission rates could be investigated. Future studies could also look at the congruence between occupational therapist recommended discharge locations and the actual discharge location of the patient. This would allow for analysis if discharge locations were related to readmission status and what percentage of discharges readmitted were against therapy staff recommendations. Additionally, future research may investigate readmitting diagnoses to determine if occupational therapy services are able to further reduce social admissions.

## Conclusion

As readmission status has become an increasingly analyzed healthcare metric, this study sought to understand the effects of occupational therapy services on a patient’s readmission status for patients with a cancer diagnosis. These results demonstrate that occupational therapy services have a significant protective association against a 30-day hospital readmission even when adjusting and accounting for multiple demographic and diagnostic attributes. These findings provide support for the implementation of occupational therapy services to patients with cancer as well as corroborate previous findings related to the benefit of occupational therapy services for the general inpatient hospital population.

## Supplementary Information

Below is the link to the electronic supplementary material.

Supplementary Material 1

## Data Availability

Datasets are available through VCU Health Systems and not publically available.
