# Supplementary material for: The effect of occupational therapy services on hospital readmission for patients with cancer in acute care settings: a retrospective data analysis
Source: J Cancer Surviv. 2024 May 31;19(6):2091–100. doi: 10.1007/s11764-024-01620-4 (PMC12546482; doi:10.1007/s11764-024-01620-4)
Supplement: Supplementary file 1 — Supplementary Material 1 [file 11764_2024_1620_MOESM1_ESM.docx]

**Table S1**

*Logistic Regression Results of OT Services and Odds of Hospital Readmission with Covariates*

|  | | B | | Sig. | Exp(B) | 95% C.I.for EXP(B) | |
| --- | --- | --- | --- | --- | --- | --- | --- |
|  |  |  |  |  |  | Lower | Upper |
| Step 1^a^ | OT Services | -.251 | | .046 | .778 | .608 | .996 |
|  | Sex | -.065 | | .614 | .938 | .730 | 1.205 |
|  | White | Reference | | - | - | - | - |
|  | Black | -.096 | .434 | | .908 | .714 | 1.156 |
|  | Asian | .197 | .674 | | 1.218 | .487 | 3.049 |
|  | Other | -.247 | .494 | | .781 | .386 | 1.583 |
|  | Non-Hispanic | Reference | - | | - | - | - |
|  | Hispanic | -.056 | .897 | | .946 | .405 | 2.207 |
|  | Age At Visit | -.012 | .025 | | .988 | .977 | .998 |
|  | Admitting Diagnosis | -.218 | .072 | | .804 | .634 | 1.019 |
|  | Gastrointestinal | Reference | - | | - | - | - |
|  | Breast | -.098 | .676 | | .907 | .573 | 1.434 |
|  | Blood/Heme | .239 | .131 | | 1.270 | .931 | 1.731 |
|  | Gynecological | -.096 | .715 | | .908 | .542 | 1.523 |
|  | Prostate/Genitourinary | -.131 | .548 | | .877 | .571 | 1.347 |
|  | Respiratory | -.438 | .011 | | .645 | .461 | .903 |
|  | Cancer Stage 1 | Reference | - | | - | - | - |
|  | Cancer Stage 2 | -.138 | .488 | | .871 | .590 | 1.286 |
|  | Cancer Stage 3 | .114 | .548 | | 1.121 | .772 | 1.627 |
|  | Cancer Stage 4 | -.020 | .908 | | .980 | .696 | 1.380 |
|  | Home with Others | Reference | - | | - | - | - |
|  | Home Alone | -.316 | .031 | | .729 | .547 | .972 |
|  | LTSS | -.401 | .249 | | .670 | .339 | 1.323 |
|  | Living-Other | .644 | .204 | | 1.904 | .706 | 5.136 |
|  | Earliest Pain Score | .001 | .962 | | 1.001 | .966 | 1.037 |
|  | Last Pain Score | .043 | .070 | | 1.044 | .996 | 1.093 |
|  | Commercial | Reference | - | | - | - | - |
|  | Corrections | -.661 | .320 | | .516 | .140 | 1.902 |
|  | Indigent | -.247 | .389 | | .781 | .445 | 1.371 |
|  | Medicaid | .060 | .729 | | 1.062 | .757 | 1.489 |
|  | Medicare | .067 | .675 | | 1.070 | .780 | 1.466 |
|  | Military | -.065 | .942 | | .937 | .160 | 5.470 |
|  | Other Insurance | -2.282 | .029 | | .102 | .013 | .791 |
|  | Self-Pay | -.387 | .574 | | .679 | .176 | 2.624 |
|  | Discharge Home | Reference | - | | - | - | - |
|  | Discharge Rehab | -.309 | .135 | | .734 | .490 | 1.100 |
|  | Discharge Home with Support | .073 | .630 | | 1.075 | .800 | 1.446 |
|  | Discharge Expired | -20.309 | .995 | | .000 | .000 | . |
|  | Discharge Hospice | -2.050 | <.001 | | .129 | .064 | .258 |
|  | Discharge AMA | .790 | .259 | | 2.204 | .559 | 8.687 |
|  | Discharge Additional hospital care | -1.267 | .234 | | .282 | .035 | 2.273 |
|  | Constant | .248 | .535 | | 1.282 |  |  |

*Note.* OT services reference to no received OT services;* OT=occupational therapy; LTSS=long-term services and supports; AMA=against medical advice. *OR=Odds Ratio; p<0.05 is considered a significant difference*

*Model adjusted for: sex, race, ethnicity, age at visit, admission diagnosis as cancer, cancer type, cancer stage, living situation prior to admission, earliest pain score, last pain score, insurance type, and discharge placement.*
